# A Canonical DREB2-Type Transcription Factor in Lily Is Post-translationally Regulated and Mediates Heat Stress Response

**DOI:** 10.3389/fpls.2018.00243

**Published:** 2018-03-08

**Authors:** Ze Wu, Jiahui Liang, Shuai Zhang, Bing Zhang, Qingcui Zhao, Guoqing Li, Xi Yang, Chengpeng Wang, Junna He, Mingfang Yi

**Affiliations:** ^1^Beijing Key Laboratory of Development and Quality Control of Ornamental Crops, College of Horticulture, China Agricultural University, Beijing, China; ^2^Department of Fruit Science, College of Horticulture, China Agricultural University, Beijing, China

**Keywords:** *Lilium longiflorum*, DREB2 transcription factor, heat stress, negative regulatory domain, alternative splicing, RCD1, BPM2

## Abstract

Based on studies of monocot crops and eudicot model plants, the DREB2 class of AP2-type transcription factor has been shown to play crucial roles in various abiotic stresses, especially in the upstream of the heat stress response; however, research on DREB2s has not been reported in non-gramineous monocot plants. Here, we identified a novel DREB2 (LlDREB2B) from lily (*Lilium longiflorum*), which was homologous to AtDREB2A of Arabidopsis, OsDREB2B of rice, and ZmDREB2A of maize. *LlDREB2B* was induced by heat, cold, salt, and mannitol stress, and its protein had transcriptional activity, was located in the nucleus, was able to bind to the dehydration-responsive element (DRE), and participated in the heat-responsive pathway of HsfA3. Overexpression of *LlDREB2B* in Arabidopsis activated expression of downstream genes and improved thermotolerance. LlDREB2B was not regulated by alternative splicing; functional transcripts accumulated under either normal or heat-stress conditions. A potential PEST sequence was predicted in LlDREB2B, but the stability of the LlDREB2B protein was not positively affected when the predicated PEST sequence was deleted. Further analysis revealed that the predicated PEST sequence lacked a SBC or SBC-like motif allowing interaction with BPMs and required for negative regulation. Nevertheless, LlDREB2B was still regulated at the post-translational level by interaction with AtDRIP1 and AtDRIP2 of Arabidopsis. In addition, LlDREB2B also interacted with AtRCD1 and LlRCD1 via a potential RIM motif located at amino acids 215–245. Taken together, our results show that LlDREB2B participated in the establishment of thermotolerance, and its regulation was different from that of the orthologs of gramineous and eudicot plants.

## Introduction

As a result of extensive fossil energy use, greenhouse gas emissions have increased year by year, leading to an irreversible trend of global warming across recent decades and continuing into the future (Grover et al., 2013; Teixeira et al., 2013). Temperatures above the normal optimum are sensed by all organisms as heat stress (HS), which can directly affect the properties of various cellular components (e.g., nucleic acid structure, protein stability, and membrane fluidity) and can therefore disrupt cellular homeostasis, causing cellular malfunction and even leading to cell death (Wang et al., 2004; Kotak et al., 2007; Wahid et al., 2007). Most food and horticultural crops are very sensitive to high temperature, which can result in decreased yield and quality (Barnabas et al., 2008; Bita and Gerats, 2013; Deryng et al., 2014). Therefore, understanding heat stress response (HSR) mechanisms is essential to improving thermotolerance and reducing the adverse influence of heat on plant growth.

Unlike animals, which can escape from adverse environments, plants, being sessile organisms, are vulnerable to climate change. Therefore, plants have evolved more complicated molecular adaptations to both survive and sustain growth in harsh environments (Kotak et al., 2007; Scharf et al., 2012). In response to various abiotic stresses, stress-induced gene expression are largely regulated by transcription factors (TFs), which play central roles in the regulation of target gene expression via specific binding to *cis*-acting elements in their promoters (Mitsuda and Ohme-Takagi, 2009; Golldack et al., 2011). One such class of TFs is DREB/CBF, which binds to DRE to activate expression of responsive genes (Yamaguchi-Shinozaki and Shinozaki, 1994; Stockinger et al., 1997; Agarwal et al., 2006; Lata and Prasad, 2011).

The DREB TFs, DREB1 and DREB2, are important APETALA2 (AP2)/ethylene responsive factor (ERF) that broadly participate in plant stress response pathways (Agarwal et al., 2017). The Arabidopsis DREB1 subgroup consists of six genes; DREB1A/CBF3, DREB1B/CBF1, and DREB1C/CBF2 are induced by low temperature stresses (Gilmour et al., 1998; Seki et al., 2001; Fowler and Thomashow, 2002); however, CBF4/DREB1D, DREB1E/DDF2, and DREB1F/DDF1 are induced by osmotic stress (Nakashima et al., 2009). Expression of DREB2 genes is also induced by osmotic stress, suggesting the existence of cross-talk between the DREB1 and DREB2 pathways. Dehydration, high-salinity, and heat also activate the expression of DREB2 genes (Liu et al., 1998; Nakashima et al., 2000; Sakuma et al., 2006b; Matsukura et al., 2010; Mizoi et al., 2013). In Arabidopsis, wheat, rice, maize, and Chrysanthemum, DREB2s also respond to low temperature (Egawa et al., 2006; Qin et al., 2007; Liu et al., 2008; Lee et al., 2010; Matsukura et al., 2010). The DREB1 and DREB2 regulons can be used to improve the tolerance of various agriculturally important crop plants to high-salinity, drought, freezing, and heat stresses by gene transfer (Bhatnagar-Mathur et al., 2007; Zhao et al., 2010; Cui et al., 2011; Mallikarjuna et al., 2011; Zhou et al., 2012). Although DREBs have been identified and characterized in various plants, including grasses, crops, legumes, and Arabidopsis, and their involvement in stress tolerance has been established (Mizoi et al., 2012), studies of DREBs in non-gramineous monocot plants are scarce.

Lily (*Lilium* spp.) is an important horticultural crop accounting for a large part of the worldwide cut-flower market (Xin et al., 2010). Lily is generally well adapted to cool climatic conditions of about 18–22°C. However, high temperatures may cause stagnation of vegetative growth, diminished cut-flower quality, and degeneration of the bulb (Gong et al., 2014). In summer, most parts of China suffer high temperatures which are harmful to lily production; therefore, increasing thermotolerance is an important objective for improving the field performance of lily. As a non-gramineous monocot, lily is an ancient species very different from Gramineae and eudicot species. Research on lily can therefore improve our knowledge of HS response mechanisms in different plants.

DREB2s are reported to play crucial roles in HS, and their overexpression can improve thermotolerance of plants (Lata and Prasad, 2011; Li et al., 2014). In Poaceae, post-transcriptional control via alternative splicing is a key regulatory manner of DREB2 TFs; however, this similar alternative splicing regulation is not found in other plant species (Shen et al., 2003; Qin et al., 2007; Matsukura et al., 2010). Post-translational regulation with a negative regulatory domain (NRD) is important for DREB2 regulation in Arabidopsis and soybean; whether this regulation is common among other plant species is also unclear (Sakuma et al., 2006a; Mizoi et al., 2013). In this study, a novel *DREB2* (*LlDREB2B*) from lily was isolated and characterized. When overexpressed in Arabidopsis, LlDREB2B could induce expression of downstream genes involved in heat response and enhance thermotolerance. The regulatory mechanism of *LlDREB2B* in lily was very different from that of the homologous genes *AtDREB2A* in Arabidopsis or *OsDREB2B* in rice.

## Materials and Methods

### Plant Materials, Growth Conditions, and Stress Treatments

The *Lilium longiflorum* hybrid ‘White heaven,’ which showed better thermotolerance than other cultivars, was used. Lily plantlets were cultured on MS medium at 22°C in a culture room with a photoperiod of 16 h light and 8 h dark. For analysis of *LlDREB2B* gene function, the model plant *Arabidopsis thaliana* (Col-0) was selected since its genetic transformation methods are well established. Arabidopsis plants were grown in plastic cups containing a sterile rooting mixture under controlled conditions (22/16°C, 16 h light, and 8 h dark). Seeds of *Nicotiana benthamiana* were planted in a sterile rooting mixture and cultured under the same conditions.

For heat treatments, 2-week-old, healthy lily plantlets (in bottles, diameter: 6 cm, height: 12 cm) of uniform size (bulb perimeter: 1.5–2.0 cm; number of leaves: 3–5; height: 6–8 cm) were exposed to different temperatures (16, 22, 28, 32, 37, 42°C) for 3 h or to 37°C for various lengths of time (0, 1, 3, 6, 12, 24, 48 h). All heat treatments were applied in a temperature-controlled incubator (DRP-9082, SUMSUNG, China) without light. For cold treatments, lily plantlets were treated for 24 h at 4°C in a refrigerator (SIEMENS, Germany). For NaCl and mannitol treatments, plantlets were transferred to double-distilled water as control or 200 mM NaCl or 400 mM mannitol for 24 h in the culture room. Each treatment was repeated three times. After treatment, leaves were collected for *LlDREB2B* expression analysis.

### Cloning *LlDREB2B* cDNA

Following the manufacturer’s instructions of an RNAprep Pure Plant kit (TIANGEN, China), total RNA extraction was performed from leaves of ‘White heaven’ incubated at 37°C for 1 h. First-strand cDNA was synthesized by using M-MLV (Takara, Japan) with an oligo dT primer. A conserved partial sequence of the *LlDREB2B* cDNA was amplified using degenerate primers (F: GGBTCRAAGAARGGNTGTATGAA and R: ATMTCAGMAACCCMTTTVCCCCA) based on the AP2 domain of deduced DREB2 polypeptides, then cloned into pMD-18T (Takara, Japan) for sequencing. Rapid amplification of cDNA ends (RACE) was performed with a 5′- and 3′-one-step Full Race kit (Takara, Japan). After sequencing, the full-length *LlDREB2B* was obtained. Two *LlDREB2B* variants were isolated and identified by reverse-transcription (RT)-PCR. Primers are listed in Supplementary Table S1.

### Phylogenetic Analysis and Conserved Protein Motif Prediction

ExPASy online tools^1^ were used for translation. Multiple sequence alignment of the deduced DREB amino acid sequences of different plant species was performed using Clustal-W in conjunction with BioEdit7.0 software. Phylogenetic trees were generated by the neighbor-joining method using MEGA 5.1. The potential PEST sequence was predicated using the epestfind program^2^.

### Gene Expression Analysis by Quantitative PCR in Response to Abiotic Stress

Total RNA was extracted as described above, and reverse transcription was performed with a HiScript II kit (Vazyme, China). Real-time quantitative PCR (qRT-PCR) (refer to the method of Gong et al., 2014) was used to determine expression levels. Lily *18S rRNA* served as a quantification control. Primers are listed in Supplementary Table S2.

### Promoter Isolation and Sequence Analysis

Following the manufacturer’s instructions of a Plant Genprep DNA kit (Zomanbio, China), genomic DNA was extracted from lily leaves. The *LlDREB2B* promoter was isolated using hiTAIL-PCR (Liu and Chen, 2007). A fragment of 1283 bp upstream from the start ATG of *LlDREB2B* was isolated and identified. The *cis*-elements in the promoter were analyzed by software online tool ^3^.

### Transcriptional Activity Analysis of LlDREB2B in Yeast

The complete *LlDREB2B* open reading frame (ORF) was inserted between the *Eco*RI and *Pst*I sites of *pGBKT7* vector (Clontech). The recombinant plasmid was transformed into yeast strain AH109 for assay of transcriptional activity. Yeast strains harboring the GAL4 plasmid and empty plasmid were used as positive and negative controls, respectively. After incubation at 30°C for 3 days, all strains were used for β-galactosidase activity analysis. Colony-lift filter and enzyme assay procedures were performed according to Gong et al. (2014). Primers for vector construction are shown in Supplementary Table S3.

### Subcellular Localization of LlDREB2B

The *LlDREB2B* ORF (without stop codon) was amplified by primers with *Xba*I and *Kpn*I sites (Supplementary Table S3), and then cloned into *pCAMBIA1300-C-GFP* upstream of the GFP sequence to generate the LlDREB2B-GFP fusion protein. The nuclear localization signal (NLS) of *LlDREB2B* was deleted, and then the remainder of the gene cloned into *pCAMBIA1300-C-GFP*. With the freeze-thaw method, the empty and reconstructed vectors were introduced into *Agrobacterium tumefaciens* strain GV3101, respectively. Tobacco (*N. benthamiana*) leaves were infiltrated with bacteria solution for transient transformation as described previously (Gong et al., 2014). The empty vector was used as a control. A confocal laser-scanning microscope was used for GFP fluorescence detection (FV1000, Olympus, Japan).

### Yeast One-Hybrid (Y1H) Analysis

Three repeat DREs or mutant DREs (mDREs) were inserted into the *pHis2.1* vector using *Eco*RI and *Spe*I to generate *pHis2.1-3DRE* or *pHis2.1-3mDRE*. A fragment (-746 to -668) of the *LlHsfA3B* promoter was amplified by PCR, then cloned into the *Eco*RI and *Spe*I sites to generate *pHis2.1-3B-DRE*; some DREs of this fragment (shown in **Figure 5**) were mutated and cloned into the same sites to generate *pHis2.1-3B-mDRE*. Full-length *LlDREB2B* was amplified by primers harboring *Bam*HI and *Xho*I sites. The product was inserted into *pGADT7* (Clontech) to generate *pGADT7-LlDREB2B*. Primers are shown in Supplementary Table S3. The corresponding vectors were co-transformed into yeast strain Y187 to investigate binding. Successful transformants were selected by growth on SD media (Clontech) without Leu and Ura at 30°C for 3 days.

### Yeast Two-Hybrid (Y2H) Analysis

The ORFs of *AtDREB2A* and *LlDREB2B* were inserted into *pGADT7*, respectively. The ORFs of *AtDRIP1* (125-C end), *AtDRIP2* (131-C end), *AtRCD1*, *AtBPM2*, *LlRCD1*, and *LlBPM2* were inserted into *pGBKT7*, respectively. Empty vectors *pGADT7* and *pGBKT7* were used as negative controls. Primers for vector construction are shown in Supplementary Table S2. The corresponding vectors were co-transformed into yeast strain AH109 for investigation of interaction. Successful transformants were selected by growth on Leu and Trp deficient SD media at 30°C for 3 days.

### Transient Assays and Fluorescence Microscopy of Tobacco Leaf Cells

*LlDREB2B* and *LlDREB2B-D* were inserted into *pCAMBIA1300-C-GFP*, respectively. The vectors were introduced into *Agrobacterium tumefaciens* strain GV3101, and bacterial solutions (OD_600_ = 1.0) were infiltrated into tobacco leaves. Fluorescence was observed using a fluorescence microscope after 2 days (Olympus, Japan). These infiltrated tobacco leaves were harvested, and total protein was extracted using a Plant Total Protein Extraction Kit PL0601-50 (Bangfei, China) according to the manufacturer’s protocol. The target proteins were immunologically detected using an anti-GFP antibody (Sigma, United States).

### Generation of *LlDREB2B* Transgenic Arabidopsis

The *LlDREB2B* ORF was amplified by primers containing *Xba*I and *Kpn*I sites and cloned into *pCAMBIA1300* under control of a *35S* CaMV promoter. The 1283 bp promoter of *LlDREB2B* was amplified from the genome by using primers containing *Pst*I and *Bam*HI sites and cloned into *pCAMBIA1391* containing a *GUS* (β*-glucuronidase*) reporter gene. Primers are shown in Supplementary Table S2. The recombinant vectors were transformed, respectively, into 5-week-old Arabidopsis plants by using *Agrobacterium* GV3101 and the floral-dip method. Transformed seeds were selected on MS medium containing 30 mg L^-1^ hygromycin. All transgenic lines were identified by RT-PCR; T3-generation homozygous lines were selected for gene functional analysis.

### MG132 Treatment of *GFP*-Fused *LlDREB2B* Transgenic Plants

*pCAMBIA1300-C-GFP-LlDREB2B* was transformed into Arabidopsis as described above. Homozygous lines were acquired by three rounds of selection. Wild-type and transgenic plants were cultured on MS medium for 5 days, then transferred to two-layer filter paper containing 1/2 liquid MS medium under dim light conditions, followed by MG132 (Sigma, United States) treatment. After 12 h, fluorescence was observed using a fluorescence microscope (Olympus, Japan).

### GUS Activity Assay of Promoter Transgenic Lines

Histochemical staining for GUS activity assay of transgenic plants was performed following the method of Hwang et al. (2014). The 7-day-old seedlings were immersed in GUS staining solution and incubated at 37°C for 12 h. Salt and mannitol treatments lasted 12 h. Heat treatment lasted 3 h at 37°C. Cold treatment lasted 12 h at 4°C. Chlorophyll was cleared by immersing these plants in 70% ethanol for 24 h.

### Thermotolerance Test of Transgenic Arabidopsis Seedlings

Arabidopsis seeds were sterilized with 1.0% (v/v) NaClO for 15 min, then washed five times with sterile distilled water and sown onto MS medium. After vernalization for 3 days at 4°C in the dark, seeds were incubated in a culture room (22°C, 16-h light/8-h dark regime). For HS, plates containing 5-day-old seedlings (transgenic lines and wild-type) were sealed with plastic electric tape and transferred to an incubator (as shown in figures). After HS, plates were removed from the incubator and kept at 22°C with the same photoperiod; less-thermotolerant seedlings would lose green color and die, and their survival rate after a 7-day recovery was recorded.

### Expression Analysis of Downstream Genes by qRT-PCR

The 5-day-old wild-type and transgenic seedlings were used for gene expression analysis. RNA extraction and qRT-PCR were performed as described above. *AtActin2* was used as a normalization control. Primers are shown in Supplementary Table S3.

## Results

### Molecular Cloning of *LlDREB2B*

**FIGURE 1:**
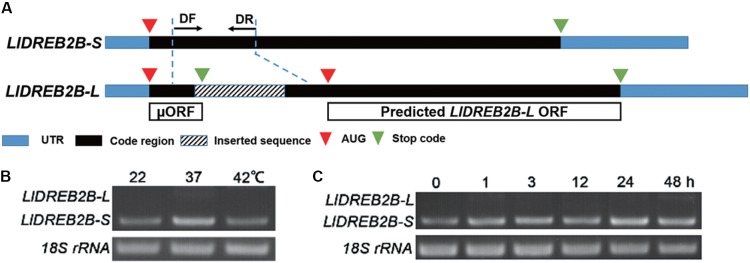
Isolation and identification of *LIDREB2B* from lily. **(A)** Schematic diagram indicating the two variants of *LIDREB2B. LIDREB2B-L* has the same nucleotide sequence as *LIDREB2B-S* except for a 198-bp sequence insertion in the codon region, which causes a frame shift and premature termination to generate a short ORF (μORF). **(B)** RT-PCR assay of transcript accumulations of *LIDREB2B-L* and *LIDREB2B-S* under different temperature treatments. Leaf samples of ‘White heaven’ were treated at 37 or 42°C for 3 h. Primers (DF and DR) were designed to amplify the two variants in one reaction as indicated in **A**. **(C)** RT-PCR assay of transcript accumulations of *LIDREB2B-L* and *LIDREB2B-S* under HS treatments of different lengths. Leaf samples of ‘White heaven’ were treated with HS at 37°C for 0, 1, 3, 12, 24, or 48 h. Primers were the same as in **B**. Bands in **B,C** were confirmed by sequencing. Three independent experiments were performed, and one representative is shown.

Two full-length *LlDREB2B* cDNAs were isolated from HS lily leaves by full RACE PCR. The products were sequenced: *LlDREB2B-S* was 1225 bp and *LlDREB2B-L* was 1423 bp, both containing a 5′-untranslated region (UTR) of 77 bp and a 3′-UTR of 236 bp. *LlDREB2B-S* had a single, continuous ORF encoding a polypeptide of 325 amino acids with a predicted molecular mass of 78.05 kDa and a predicted isoelectric point of 5.01. *LlDREB2B-L* had the same nucleotide sequence as *LlDREB2B-S* except for a 198-bp sequence insertion in the codon region, which caused a frame shift and premature termination to generate a short ORF. Ignoring the short ORF, *LlDREB2B-L* was predicated to encode a truncated DREB2 protein (**Figure 1A**). RT-PCR analysis demonstrated that *LlDREB2B-S* was the primary transcript under either HS or normal conditions, but *LlDREB2B-L* showed low-level accumulation (**Figures 1B,C**). In addition to HS, *LlDREB2B-L* also showed low-level accumulation under salt, mannitol, and cold stress (Supplementary Figure S1). As *LlDREB2B-L* lacked some amino acids and had low expression, we regarded the protein encoded by *LlDREB2B-S* as LlDREB2B.

### Sequence Analysis of LlDREB2B

**FIGURE 2:**
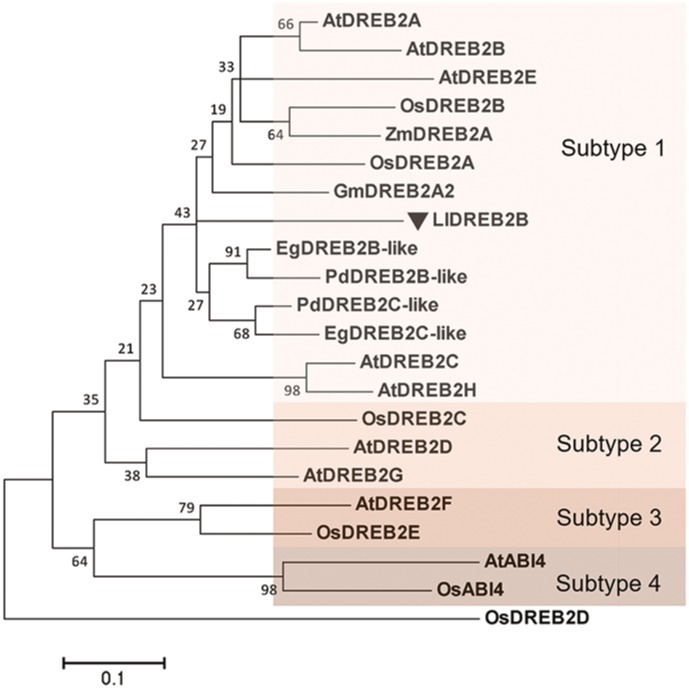
Phylogenetic analysis of LlDREB2B and related proteins. Neighbor-joining tree based on alignment of the peptide sequences of the N-terminal conserved region and the DNA-binding domain (Supplementary Figure S3). Accession numbers are shown in Supplementary Figure S3. The tree was constructed using MEGA5.1. Numbers at nodes indicate bootstrap values from 1,000 replicates. Bar, substitution rate per site. Each subtype is indicated by a colored rectangle.

The full-length amino acid sequence of LlDREB2B used as a query to perform an NCBI BLAST, the search result revealed that the deduced LlDREB2B possessed a conserved AP2 domain with a high degree of similarity to those of EgDREB2B (*Elaeis guineensis*) and PdDREB2B (*Phoenix dactylifera*) (47 and 46%, respectively). Multiple alignments with other DREB2B proteins from Arabidopsis, soybean, rice, and maize showed that LlDREB2B contained conserved CMIV-1, CMIV-2, and AP2 motifs (Supplementary Figure S2A). The conserved CMIV-3 motif was also found to be present in LlDREB2B by manual blast analysis with DREB2 homologs according to previous analysis (Supplementary Figure S2B) (Nakano et al., 2006). LlDREB2B also contained a predicted conserved NLS domain (amino acids 52–72). A neighbor-joining phylogenetic tree showed that LlDREB2B belonged to the subtype-1 group and was most closely related to EgDREB2B from the non-gramineous monocot *Elaeis guineensis* (**Figure 2**). Alignment of AP2/ERF DNA-binding domain sequences demonstrated that LlDREB2B contains the conserved valine (V) and glutamic acid (E) residues observed in other DREB2s. Interestingly, OsDREB2B and ZmDREB2A were found to have no NRD following the AP2domain; however, LlDREB2B was predicted to have a serine/threonine-rich potential PEST sequence of a NRD (amino acids 156–181) using the epestfind program^4^ (Supplementary Figure S2).

### *LlDREB2B* Is Induced by Heat, Cold, Salt, and Mannitol

**FIGURE 3:**
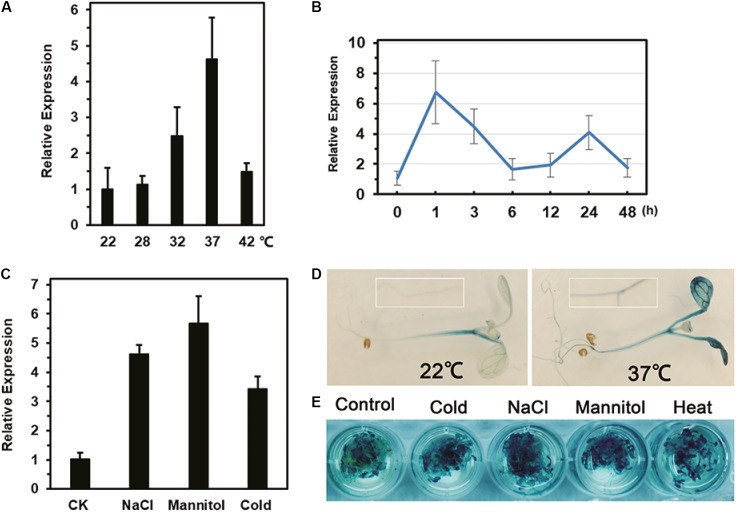
Analysis *of LlDREB2B* expression and promoter activity under different abiotic stresses. Relative expression in leaves after **(A)** 3 h under different ambient temperature treatments, **(B)** exposure to 37°C for different lengths of time, and **(C)** treatment of tissue-cultured lily seedlings by placing their roots in water (CK), salt solution (NaCl, 200 mM), or mannitol solution (400 mM) for 24 h. For cold treatments, lily seedlings were treated for 24 h at 4°C. Lily *18S rRNA* was used for data normalization. Each treatment included three plants. Bars are means ± SD of three independent experiments. **(D)** Histochemical analysis of GUS activity in 7-day-old transgenic seedlings grown under normal conditions and treated at 37°C for 3 h. **(E)** GUS analysis of seedlings treated with water (control), salt solution (NaCl, 150 mM), mannitol solution (300 mM), or cold (4°C) for 12 h, or HS (37°C) for 3 h. Three independent experiments were performed, and one representative is shown.

Compared with expression at 22°C, the expression of *LlDREB2B* was up-regulated by c. 2.5-fold and 4.6-fold after 3 h of treatment at 32 and 37°C, respectively (**Figure 3A**). When exposed to 37°C, *LlDREB2B* was rapidly induced; expression peaked after 1 h and then gradually decreased, but was enhanced again after 24 h of treatment (**Figure 3B**). In addition, *LlDREB2B* was induced by cold, salt, and mannitol stress (**Figure 3C**).

### Activity Assay of the *LlDREB2B* Promoter

GUS activity in transgenic seedlings showed that the *LlDREB2B* promoter had basal activity under normal conditions, but the activity was greatly elevated by HS in both leaves and roots (**Figure 3D**). GUS activity was also enhanced after cold, salt, or mannitol treatment (**Figure 3E**). Surprisingly, there were no conserved HS elements (HSEs, nGAAnnTTCn) in the *LlDREB2B* promoter; however, many *cis*-elements associated with dehydration stress and the ABA pathway were present (Supplementary Table S4).

### LlDREB2B Localizes to the Nucleus and Has Transactivation Activity

**FIGURE 4:**
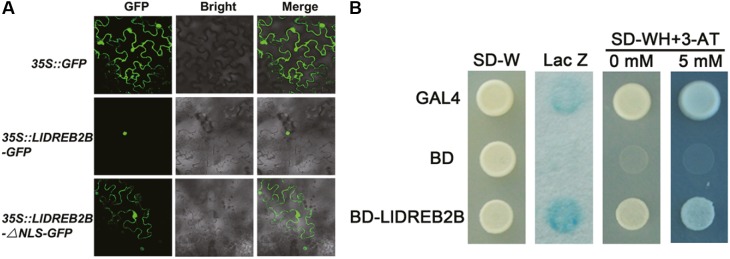
Subcellular localization and transactivation assay of LlDREB2B protein. **(A)** Transient expression profiles of LlDREB2B in tobacco leaves. Confocal microscopy of tobacco leaf cells transfected with *LIDREB2B* or *LIDREB2B-ANLS* fused to the N-terminal *GFP* reporter gene controlled by the *35S* promoter. Empty *GFP* vector served as a negative control. **(B)** Transactivation activity of LlDREB2B in yeast cells. GAL4 and BD were used as positive and negative controls, respectively. SD-Trp (SD-W) medium was used to detect transformation, SD-Trp/-His (SD-WH) medium with or without 3-AT (3-amino-1,2,4-triazole, a competitive inhibitor of the HIS3 protein) was used to examine the growth of transformants, and X-gal staining was used to detect the p-galactosidase activity of transformed yeast cells. Three independent experiments were performed, and one representative is shown.

Fluorescence of the fusion protein LlDREB2B-GFP was observed in the nucleus (**Figure 4A**). Protein sequence alignment showed that LlDREB2B contained a conserved NLS domain (Supplementary Figure S2A). To investigate this NLS function, LlDREB2B with the NLS deleted was fused with GFP at its C-terminal. After transient expression, fluorescence was distributed in the nucleus and cytoplasm, indicating that the predicted NLS of LlDREB2B could guide the protein to the nucleus (**Figure 4A**). The *pGBKT7* vectors required for transactivation analysis were transformed into yeast AH109. Yeast cells containing LlDREB2B could grow well on –WH plates and catalyzed degradation of β-galactosidase, indicating that LlDREB2B had transactivation activity in yeast cells (**Figure 4B**).

### LlDREB2B Can Bind to DREs

**FIGURE 5:**
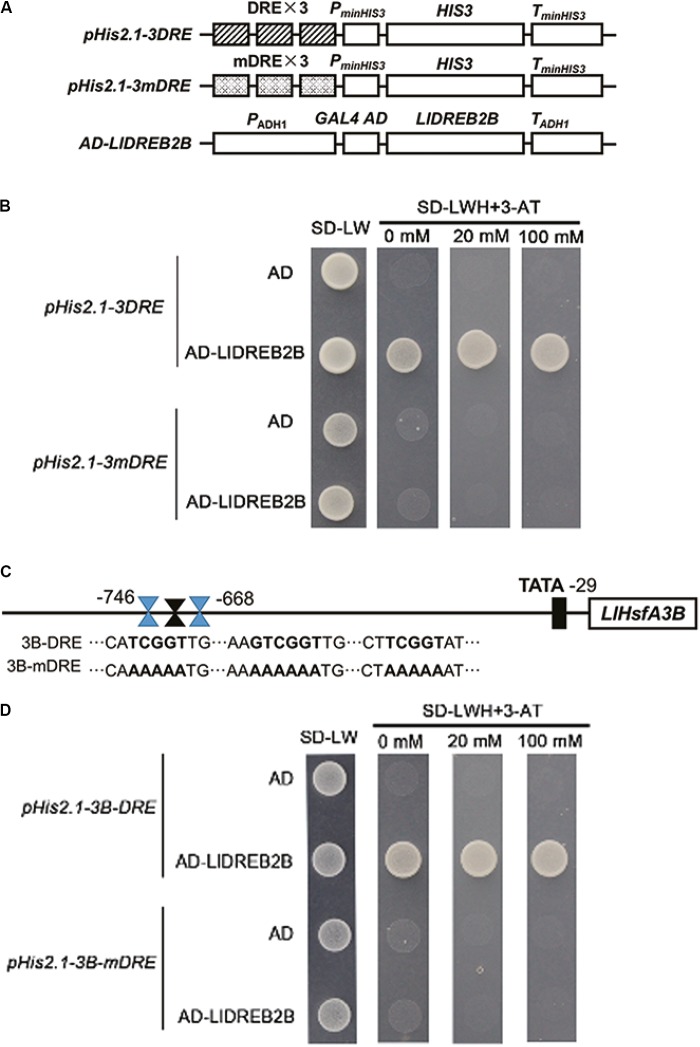
Analysis of the DRE binding ability of LlDREB2B. **(A)** Schematic diagrams of the required vectors for yeast one-hybrid assay. The *pHis2.1* vector contained three tandem repeats of a DRE (TATACTACCGACATGAGTTC) or mutant DRE (TATACTAAAAAAATGAGTTC) sequence and the HIS reporter gene. **(B)** Yeast strain Y187 was co-transformed with bait (*pHis2.1-3DRE* or *pHis2.1-3mDRE*) and a prey (*pGADT7* or *pGADT7-LlDREB2B*) construct. Interaction between bait and prey was determined by cell growth on SD medium lacking Trp, Leu, and His (SD-WLH) and containing the indicated concentrations of 3-AT. **(C)** Sequences of the *LlHsfA3B* promoter and its DRE-mutated version. **(D)** Yeast strain Y187 was co-transformed with bait (*pHis2.1-3B-DRE* or *pHis2.1-3B-mDRE*) and a prey (*pGADT7* or *pGADT7-LlDREB2B*) construct. Interaction between bait and prey was determined by cell growth on SD-WLH containing the indicated concentrations of 3-AT.

Yeast cells transformed with *pGADT7-LlDREB2B* and *pHis2.1-3DRE* grew well on –LWH plates and even with 20 mM 3-amino-1,2,4-triazole (3-AT). However, when the DREs were mutated to mDREs, yeast cells could not grow on –LWH plates (**Figures 5A,B**). This indicated that LlDREB2B possessed the ability to bind DREs as a general AP2/ERF TF. According to previous reports, AtDREB2A and AtDREB2C play regulatory roles in the upstream of Arabidopsis AtHsfA3 by directly binding to the DRE located in its promoter (Schramm et al., 2008; Yoshida et al., 2008; Chen et al., 2010). Here, we also observed that DREs were present in the *LlHsfA3B* promoter (**Figure 5C**). The fragment (-746 to -668) containing DRE was isolated and its interaction with LlDREB2B was examined; the result showed that LlDREB2B could bind to this fragment in yeast cells, suggesting that LlDREB2B is involved in the regulatory pathway of HsfA3 (**Figures 5C,D**). DREs were also predicted in *HsfA3* orthologs belonging to different species, such as rice, maize, tomato, and oil palm, which suggested the DREB2-HsfA3 pathway might be conserved in plants (Supplementary Table S5).

### Predicted NRD Regulation of LlDREB2B

**FIGURE 6:**
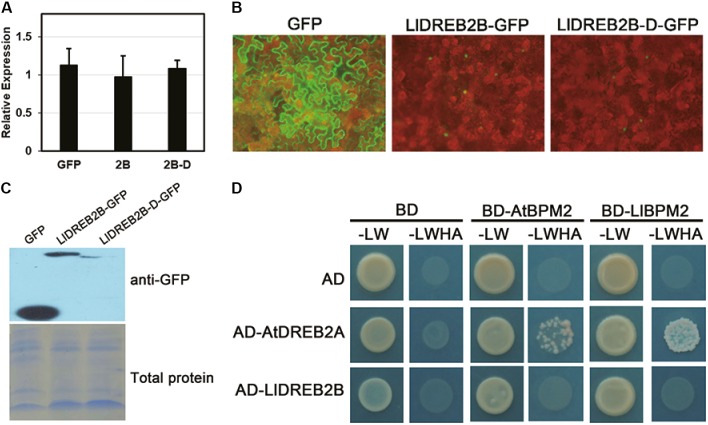
Analysis of the predicted NRD regulation of LlDREB2B. **(A)** Determination of transcript level after transient expression. Premiers targeting *GFP* were used for expression detection. Tobacco *Nt Ubiquitin* was used for data normalization. Bars are means ± SD of three biological repeats. **(B)** Fluorescence signals of GFP, LlDREB2B-GFP, and LlDREB2B-D-GFP. **(C)** Western blot assay of protein accumulation in tobacco leaves with transient expression. **(D)** Interaction of LlDREB2B or AtDREB2A with LlBPM2 and AtBPM2 determined by yeast cell growth on SD medium lacking Leu, Trp, His, and Ade (SD-LW HA).

Protein sequence analysis predicted that LlDREB2B had a potential PEST sequence located at amino acids 156–181 (Supplementary Figure S1). Previous studies have identified the region containing the PEST sequence as the NRD of DREB2, which can contribute to negative regulation of protein stability and reduce adverse effects on growth and development (Mizoi et al., 2013). To determine whether this region could also regulate LlDREB2B, we artificially deleted amino acids 150–186 to form the LlDREB2B-D protein. Stable protein was detected by transient expression in tobacco leaves. *LlDREB2B* and *LlDREB2B-D* showed a similar accumulation level in tobacco leaves (**Figure 6A**); however, the LlDREB2B-D-GFP fluorescence signal was weaker than that of LlDREB2B-GFP (**Figure 6B**), and the protein accumulation of LlDREB2B-D was also lower than that of LlDREB2B (**Figure 6C**), indicating that the predicated NRD could not negatively control the stability of LlDREB2B unlike the case for AtDREB2A. LlDREB2B-D was not more stable than LlDREB2B; instead, to our surprise, LlDREB2B-D appeared to be more unstable than LlDREB2B. In Arabidopsis, the NRD of AtDREB2A interacts with BPMs (BTB/POZ AND MATH DOMAIN proteins), which are substrate adaptors of the Cullin3 (CUL3)-based E3 ligase and promote AtDREB2A entering into the ubiquitin-mediated degradation pathway (Morimoto et al., 2017). LlBPM2, an ortholog of AtBPM2, was isolated from lily; Y2H assay showed that LlDREB2B could not interact with AtBPM2 or LlBPM2 (**Figure 6D**). These results indicated that the predicted NRD of LlDREB2B might not have the same negative regulatory function as the NRD of AtDREB2A. Analysis of the PEST sequence of the predicted NRD suggested that this region lacked a functional SBC (ϕ-π-S-S/T-S/T; ϕ, non-polar; π, polar) or SBC-like (ϕ-π-S-X-S/T; ϕ, non-polar; π, polar; X, any amino acid) motif (Supplementary Figure S1A); the SBC or SBC-like motif is required for interaction with BPMs and the conserved negative regulatory function.

### LlDREB2B Interacts With RCD1

**FIGURE 7:**
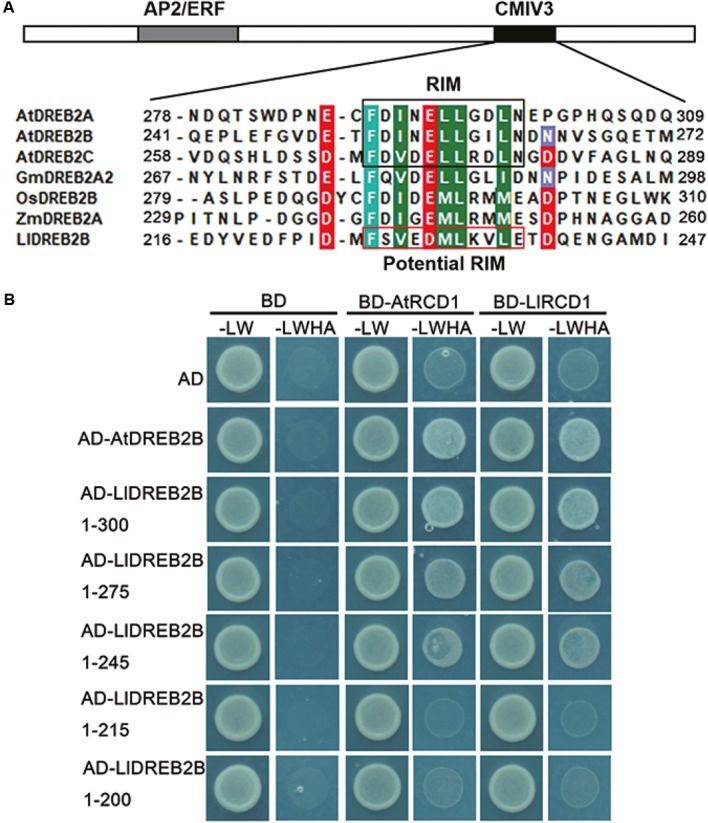
RCD1 interacts with LlDREB2B. **(A)** Alignment of the CMIV-3 sequences of different DREB2s showing conserved amino acids. The conserved RIM motif is shown in black box; LlDREB2B has a potential RIM motif (red box). **(B)** Interaction of different regions of LlDREB2B with LlRCD1 and AtRCD1 determined by yeast cell growth on SD medium lacking Leu, Trp, His, and Ade (SD-LW HA) and containing 10 mM 3-AT to repress the self-activation.

Previous studies have shown that AtRCD1 (RADICAL-INDUCED CELL DEATH 1) also inhibits AtDREB2A activity at room temperature by interaction, whereas AtRCD1 degrades rapidly and releases AtDREB2A at high temperatures (Vainonen et al., 2012). The RIM motif (FDXXXLLXX[ILMV][END]) located in the CMIV-3 box of DREB2s is central for the interaction of DREB2s with RCD1. Protein sequence analysis revealed that LlDREB2B had a potential RIM motif (FSVEDMLKVLE) in the CMIV-3 box, and Y2H assay showed LlDREB2B could interact with LlRCD1 (a RCD1 ortholog of lily) or AtRCD1. By deletion assay, it was observed that amino acids 215–245 were important for this interaction; the potential RIM motif was included in this region, which suggested the potential RIM motif has a similar function as RIM in DREB2s of Arabidopsis (**Figure 7**).

### LlDREB2B Interacts With AtDRIP1 and AtDRIP2

In addition to the stability of AtDREB2A being regulated through the NRD, AtDREB2A also interacts with the E3 ubiquitin ligases AtDRIP1 and AtDRIP2 (DREB2A-INTERACTING PROTEIN 1 and 2) prompting AtDREB2A to enter the process of 26S proteasome-mediated proteolysis. Y2H assay showed that LlDREB2B interacted with AtDRIP1 and AtDRIP2 (Supplementary Figure S4). Following treatment with the proteasome inhibitor MG132, the GFP fluorescence signal in roots of *LlDREB2B-GFP* transgenic plants was increased, which indicated LlDREB2B protein accumulation was elevated in the transgenic plants after MG132 treatment (Supplementary Figure S5). These results suggested LlDREB2B was regulated at the post-translation level in an ubiquitin/proteasome-dependent manner.

### Overexpression of *LlDREB2B* Causes Growth Defects at the Germination and Seedling Stages

**FIGURE 8:**
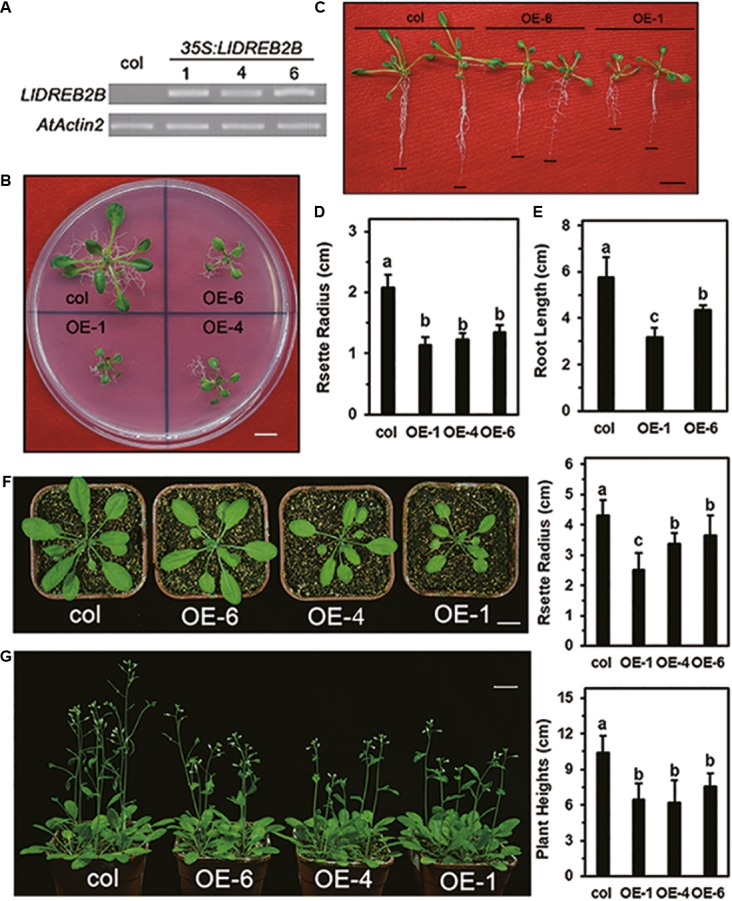
Growth of transgenic *Arabidopsis* plants expressing wild-type *LlDREB2B* under control of the *35S* promoter. **(A)** RT-PCR analysis of transgenic *Arabidopsis* lines. *AtActin2* was used as a control. **(B)** Seedlings of wild-type and transgenic lines grown on MS medium for 3 weeks. **(C)** Seedlings were grown for 7 days under normal conditions and then grown on vertically oriented MS medium for an additional 9 days. Black lines indicate root tips; Bar = 1 cm. Ten plants of each line were tested; one representative picture is shown. **(D)** Rosette radii of the plants shown in **B** were counted. **(E)** Root growth of the plants shown in **D** were counted. Bars are means ± SD of the tested plants. Different letters indicate significant differences among these lines (Student–Newman–Keuls test, *P* < 0.05). **(F)** The 2-week-old seedlings were transferred from agar plates to soil for 14 days. Bar = 1 cm. Plant rosette radii are shown to the right. Ten plants of each line were tested; one representative picture is shown. Bars are means ± SD of the tested plants. Different letters indicate significant differences among these lines (Student–Newman–Keuls test, *P* < 0.05). **(G)** The 2-week-old seedlings were transferred from agar plates to soil for 21 days. Bar = 1 cm. Plant heights are shown to the right. Eighteen plants of each line were tested; one representative picture is shown. Bars are means ± SD of the tested plants. Different letters indicate significant differences among these lines (Student–Newman–Keuls test, *P* < 0.05).

To test the function of LlDREB2B *in vivo*, transgenic Arabidopsis expressing *LlDREB2B* under the control of the *35S* promoter were generated (**Figure 8A**). Transgenic plants were grown on MS plates for 2 weeks and showed significant growth defects with a small rosette size (**Figures 8B,D**). Root growth of the transgenic plants on vertically placed agar plates was also significantly decreased (**Figures 8C,E**). After transplantation, growth of transgenic plants was also reduced compared with that of wild-type plants (**Figures 8F,G**).

### Overexpression of *LlDREB2B* Enhances the Thermotolerance of Transgenic Plants

**FIGURE 9:**
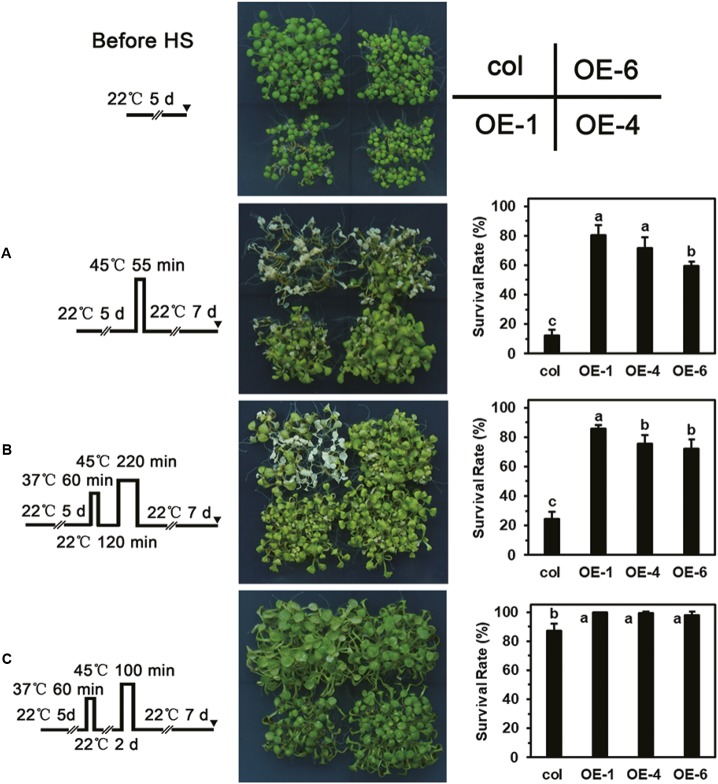
Thermotolerance test of transgenic plants. Phenotypes of wild-type and transgenic (OE-1, OE-4, and OE-6) seedlings following treatment with the HS regimes **(A–C)** shown to the far left. **(A)** Five-day-old seedlings were directly exposed to 45°C to detect BT. **(B)** Five-day-old seedlings were first treated with a non-lethal temperature of 37°C for 60 min, followed by recovery for 2 h at 22°C, and then subjected to 45°C to detect ATSR. **(C)** Three-day-old seedlings were treated at 37°C for 60 min, then cultured under 22°C for 2 days, then exposed to 45°C to detect ATLR. Wild-type and transgenic plants were photographed, and survival rate was measured after 7 days of HS. Bars are means ± SD of three independent experiments. One representative result is shown. Each treatment included over 30 seedlings of each line. Different letters indicate significant differences among these lines (Student–Newman–Keuls test, *P* < 0.05).

**FIGURE 10:**
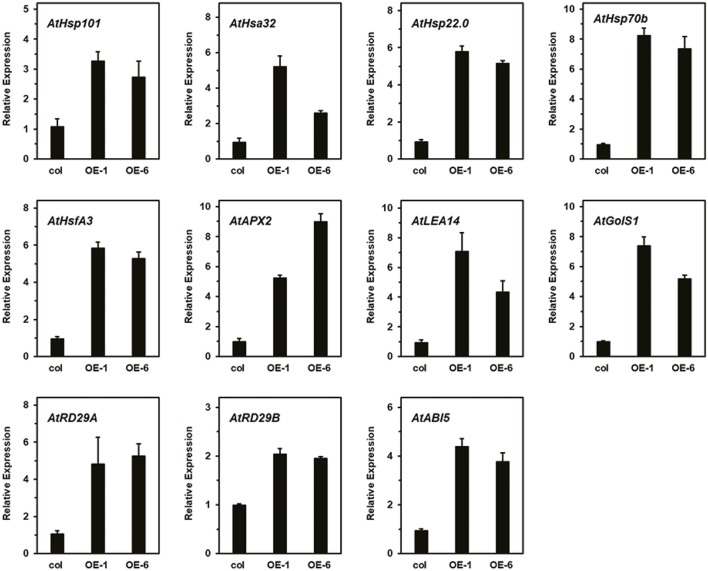
Relative expression level of AtDREB2A target genes, HS-responsive genes, and ABA-related genes in transgenic plants. Raw data were normalized using *AtActin2* as an internal reference. Data represent means ± SD of three independent experiments.

As showing in the **Figure 9**, different HS patterns were designed for the thermotolerance test. The 5-day-old seedlings were directly exposed to 45°C to detect basal thermotolerance (BT); transgenic plants showed better BT than wild-type plants, with higher survival rates (**Figure 9A**). For the detection of acquired thermotolerance (AT) after short-time recovery (ATSR), 5-day-old seedlings were first treated with a non-lethal temperature of 37°C for 60 min, followed by recovery for 2 h at 22°C, and then subjected to 45°C. After 7 days, the transgenic plants had grown better than the wild-type plants (**Figure 9B**). Three-day-old seedlings were treated with 37°C for 60 min, then cultured at 22°C for 2 days, followed by exposure to 45°C to detect acquired thermotolerance after long-time recovery (ATLR). After 7 days, the transgenic plants showed better ATLR with higher survival rates (**Figure 9C**). These results demonstrated that overexpression of *LlDREB2B* could improve BT and AT of Arabidopsis. We then measured the expression levels of HS response pathway genes in the transgenic plants under normal conditions. Transcripts of the AtDREB2A target genes *AtRD29A*, *AtRD29B*, *AtLEA14*, *AtHsfA3*, and *AtHsp70b* accumulated in transgenic plants. The expression of other genes, such as *AtHsp101*, *AtHsa32*, *AtHsp22.0*, *AtGolS1*, *AtABI5*, and *AtAPX2*, was also increased (**Figure 10**). The enhanced expression of these genes in transgenic plants might contribute to the stronger thermotolerance.

## Discussion

DREB2 TFs, as members of the AP2 family, are known to control plant responses to abiotic stresses such as freezing, cold, salt, osmotic stress, drought, and heat (Shinozaki et al., 2003; Nakashima and Yamaguchi-Shinozaki, 2006; Lata and Prasad, 2011). Their overexpression can significantly improve the stress tolerance of transgenic plants (Sakuma et al., 2006a; Qin et al., 2007; Matsukura et al., 2010; Mizoi et al., 2013). Owing to the crucial role of DREB2s, they have been widely and deeply studied in the eudicot model plant Arabidopsis and the crops rice, wheat, and maize, but their study in non-gramineous monocot plants has not been reported so far (Sakuma et al., 2006a,b; Qin et al., 2007, 2008; Kobayashi et al., 2008; Matsukura et al., 2010; Vainonen et al., 2012). In this study, we identified and characterized a heat-inducible DREB2 TF from lily, LlDREB2B, which had a typical AP2 domain with transcriptional activity, was located in the nucleus, and had the ability to bind to DREs (**Figures 2–5**).

DREB2-type TFs in rice and Arabidopsis have been classified into four subtypes (Matsukura et al., 2010). Based on expression patterns in response to stresses, together with phylogenetic analysis and peptide sequences, the homologous lily LlDREB2B is considered to be critical members of the DREB2-type subtype-1 family (**Figure 2**). In Arabidopsis, the subtype-1 DREB2s respond to drought, salt, and heat, but not cold stress (Lata and Prasad, 2011); in lily, LlDREB2B can be also induced by low temperature (**Figure 3**), suggesting it maybe participate in the low temperature response, as also observed in soybean, rice, and maize (Mizoi et al., 2013). The transcription of AtDREB2A under dehydration and heat stress is independently regulated by the different regions in its promoter. The region responsible for dehydration inducibility contains two essential elements, a coupling element3-like sequence and an ABA-responsive element, while a HSE in another region that binds with heat stress transcription factors (Hsfs) is necessary for the inducibility of AtDREB2A in response to HS (Kim et al., 2011; Yoshida et al., 2011; Huang et al., 2016). The promoter of LlDREB2B contained many *cis*-elements involved in ABA- and dehydration-responsive pathways, but the conserved HSE was absent (Supplementary Table S4), suggesting that expression of *LlDREB2B* may be independent of Hsfs.

In Arabidopsis, AtDREB2A has been identified as one of the primary regulators of drought and heat responses (Sakuma et al., 2006b). It is not only regulated at the transcriptional level, but also through protein stability; AtDREB2A was observed to be degraded by the ubiquitin-proteasome under non-stress conditions; but it can be stabilized through treatment with MG-132 (a proteasome inhibitor), and also by HS. AtDREB2A is mainly regulated post-translationally, with three kinds of proteins interacting with AtDREB2A to affect its accumulation and function (Qin et al., 2008; Vainonen et al., 2012; Morimoto et al., 2017). AtDRIP1 and AtDRIP2 are C3HC4 RING domain-containing proteins identified as AtDREB2A interactors that function as E3 ubiquitin ligases. Through the acceleration of 26S proteasome-mediated AtDREB2A proteolysis, AtDRIP1/2 negatively regulate the expression of AtDREB2A downstream genes (Qin et al., 2008). In this study, LlDREB2B could interact with AtDRIP1 and AtDRIP2, and its protein stability was increased by MG132 treatment (Supplementary Figures S4, S5), which suggests that LlDREB2B can be regulated by 26S-proteasome-mediated degradation. AtRCD1 belongs to a small plant-specific SRO (SIMILAR TO RCD ONE) protein family with six members in Arabidopsis; AtRCD1 interacts with AtDREB2A, and the interaction contributes to the control of AtDREB2A accumulation. A RIM motif in the CMIV-3 box has been confirmed as important, but not sufficient, to mediate the interaction of AtDREB2A with AtRCD1 (Vainonen et al., 2012). A similar RIM motif was also found in LlDREB2B, and it played a crucial role for the interaction of RCD1 (**Figure 7**). These results indicated the LlDREB2B protein stability could be post-translationally regulated by RCD1.

The protein level of AtDREB2A is also regulated by protein degradation via its NRD, which contains a serine/threonine-rich PEST sequence. The NRD is required for AtDREB2A interaction with RING E3 ligases, and deletion of NRD leads to stabilization of AtDREB2A (Sakuma et al., 2006a; Qin et al., 2008). Recently, BPMs have been found to interact with the NRD of AtDREB2A. They function as substrate adaptors of the CUL3-based E3 ligase, with the SBC or SBC-like motif of the NRD required for interaction. This interaction seriously reduced the stability of AtDREB2A; double knockout of DRIP1/2 or knockout of RCD1 was found to only partially enhance the stability of DREB2A, suggesting that BPMs are the major regulators of AtDREB2A protein stability (Morimoto et al., 2017). Transgenic plants overexpressing AtDREB2A show no any obvious phenotypic changes or improvement of dehydration tolerance; however, overexpressing AtDREB2A-CA, a constitutively active form by removal of the NRD, resulted in severe growth defects and significantly enhanced dehydration stress (Sakuma et al., 2006a). In soybean, GmDREB2A2 is a functional ortholog of AtDREB2A that plays roles in abiotic stress responses, and its activity is also negatively regulated by a PEST-like sequence in a NRD-mediated manner similar to that of AtDREB2A (Mizoi et al., 2013). Nevertheless, the NRD is not found in functional DREB2 orthologs of the grass family (Qin et al., 2007; Matsukura et al., 2010). To our surprise, although lily is a monocot plant closely related to the Poaceae, a potential PEST sequence was predicted in LlDREB2B. Protein stability, however, was negatively affected by deletion of the predicated NRD. It seemed that this region was important for the increased stability of LlDREB2B rather than decreasing its stability; we therefore speculated that a potential co-factor might interact with this region to positively affect its accumulation. In addition, LlDREB2B could not interact with LlBPM2 or AtBPM2, possibly due to the absence of a functional SBC or SBC-like motif (**Figure 6**), which also implied a different function of the predicated region. Transgenic plants overexpressing wild-type *LlDREB2B* exhibited growth retardation (**Figure 8**), which was also observed in Arabidopsis plants overexpressing *OsDREB2B* or *ZmDREB2A*, but not in transgenic plants overexpressing wild-type *AtDREB2A* or *GmDREB2A2* (Mizoi et al., 2013). This may be because LlDREB2B, OsDREB2B, and ZmDREB2A are not strictly regulated at the post-translational level by BPMs, unlike AtDREB2A and GmDREB2A2 (Morimoto et al., 2017).

Different from Arabidopsis and soybean, post-transcriptional control by alternative splicing is a key regulatory mechanism of DREB2-type TFs in the grass family (Mizoi et al., 2013). Under normal growth conditions, the major transcripts contain an intron sequence that is incompletely spliced because of a frame shift to form a short ORF, which does not encode a functional protein. Under stress conditions, the intron is completely spliced, producing the full-length, functional protein. The non-functional transcript is more abundant than the functional transcript during non-stress conditions; however, under environmental stress, functional transcripts are accumulated (Egawa et al., 2006; Qin et al., 2007; Matsukura et al., 2010). *AtDREB2B* is also reported to undergo alternative splicing by intron retention, but the splice occurs under severe HS; the splice variant does not exist under normal conditions (Liu et al., 2013). The alternative splicing of *AtDREB2A* occurs in the second exon with an alternative 3′ site under HS conditions. The RIM sequence of the CMIV-3 box is spliced, preventing interaction with RCD1, and increasing its accumulation under HS (Vainonen et al., 2012). We also isolated two variants of *LlDREB2B* with the full RACE method. The non-functional transcript *LlDREB2B-L* contained inserted sequence similar to the non-functional transcripts of *OsDREB2B* and *ZmDREB2A*; however, the transcript type *LlDREB2B* was hardly affected by HS, with functional transcript *LlDREB2B-S* accumulating at either high temperature or room temperature (**Figure 1**). Results of the phylogenetic analysis indicated that LlDREB2B is distantly related to the DREB2s of the grass family and clusters on the same branch as those from non-gramineous monocot plants of the palm and banana families (**Figure 2**). These results suggest that regulation of LlDREB2B may be very different from that of its orthologs OsDREB2B and ZmDREB2A.

Based on previous studies and our results, it seems that alternative splicing of DREB2s can occur in different plants, including the rice, maize, and wheat of the grass family, the eudicot Arabidopsis, and the non-gramineous monocot lily, but the functional regulation of this splicing primarily depends on the species; only grass plants accumulate abundant non-functional transcripts under normal conditions (Egawa et al., 2006; Qin et al., 2007; Matsukura et al., 2010; Liu et al., 2013). The alternative splicing of *DREB2s* is important for grass plants to repress functional protein accumulation, because high levels of DREB2s cause growth defects; by contrast, DREB2 protein levels in Arabidopsis are mainly controlled by a NRD-mediated degradation pathway. LlDREB2B was not regulated by alternative splicing or NRD-mediated degradation, but DRIP1/2 and RCD1 could modulate the stability of LlDREB2B, which suggested these two mechanisms of regulation might be conserved in different plant species. Our results also showed the predicated PEST sequence was important for the stability of LlDREB2B, which suggested an appropriate accumulation of LlDREB2B might be required for the normal growth of lily.

DREB2s have been extensively reported to be involved in the establishment of thermotolerance and play an important role upstream of the HS response (Agarwal et al., 2006; Lata and Prasad, 2011; Nakashima et al., 2014). Overexpression of *AtDREB2A* or *AtDREB2C* improves the thermotolerance of transgenic plants (Sakuma et al., 2006b; Lim et al., 2007), and *LlDREB2B*-overexpressing plants also showed enhanced thermotolerance (**Figure 9**). AtDREB2A and AtDREB2C can directly bind to DREs in the *AtHsfA3* promoter, activating *AtHsfA3* expression under HS (Schramm et al., 2008; Yoshida et al., 2008; Chen et al., 2010). The DREs can also be observed in promoters of HsfA3 orthologs of different plant species, such as rice, maize, tomato, and oil palm, which suggests the DREB2-HsfA3 regulatory pathway is conserved in plants. In our previous study, we identified at least two homologous HsfA3s in lily, LlHsfA3A and LlHsfA3B, and conserved DREs were only found in the *LlHsfA3B* promoter but not the *LlHsfA3A* promoter (unpublished data). Y1H assay confirmed that LlDREB2B could bind to DREs in the *LlHsfA3B* promoter (**Figure 5**). Overexpression of *LlDREB2B* in Arabidopsis could also induce *AtHsfA3* expression. Simultaneously, some target genes of AtDREB2A were also induced (**Figure 10**). These results indicate that LlDREB2B has a conserved function with AtDREB2A.

In this study, we isolated and identified a new *DREB2-*subtype 1 gene (*LlDREB2B*) from lily, which has a different regulatory mechanism from those of the Gramineae and eudicots. *LlDREB2B* has transcriptional activity, is induced by many abiotic stresses, and can improve the basal and acquired thermotolerance of Arabidopsis by its overexpression.

## Author Contributions

ZW, JH, JL, SZ, and MY prepared the plant materials and designed the experiments. ZW, SZ, GL, CW, QZ, and JL conducted the experiments. ZW took the photographs. ZW and XY analyzed the data, and ZW wrote the manuscript. All authors read and approved the manuscript.

## Conflict of Interest Statement

The authors declare that the research was conducted in the absence of any commercial or financial relationships that could be construed as a potential conflict of interest.

## Footnotes

1 http://au.expasy.org/tools/

2 http://emboss.bioinformatics.nl/cgi-bin/emboss/epestfind

3 https://sogo.dna.affrc.go.jp/cgi-bin/sogo.cgi?lang=en&pj=640&action=page&page=newplace

4 http://emboss.bioinformatics.nl/cgi-bin/emboss/epestfind
