# Supplementary material for: A Canonical DREB2-Type Transcription Factor in Lily Is Post-translationally Regulated and Mediates Heat Stress Response
Source: Front Plant Sci. 2018 Mar 8;9:243. doi: 10.3389/fpls.2018.00243 (PMC5852537; doi:10.3389/fpls.2018.00243)
Supplement: Supplementary file 1 [file Table_1.DOCX]

Supplemental Information Table S1. Primers used for RT-PCR detection

| *LlDREB2B-S* and *LlDREB2B-L* | F: ACGATGGTGAAGCAAGAGGACTCGGAG  R: CACTTTCTGAGGCGCGTTCCAGTTGAC |
| --- | --- |
| *18S rRNA* | F: AGTTGGTGGAGCGATTTGTCT  R: CCTGTTATTGCCTCAAACTTCC |

Supplemental Information Table S2. Primers used for qPCR.

| *LlDREB2B* |  | F: CTTTGCAGGGAGGGAGCTTGTTCT  R: ACTAGCAGCATACTAGCCTAATCCCT |
| --- | --- | --- |
| *18S rRNA* |  | F: AGTTGGTGGAGCGATTTGTCT  R: CCTGTTATTGCCTCAAACTTCC |
| *AtGolS1* | *At2g47180* | F: AGCCGTTCATCACCGCTCTTAC  R: ACTCCTGGCAACATTCAAGCAG |
| *AtHsp22.0* | *At4g10250* | F: ACTACTCCAGGCAGCTTGCTA  R: CTTGAATGGATCAGGGAACC |
| *AtHsp70b* | *At1g16030* | F: TGCACGATGTTGTTCTGGTT  R: GCAAAAGCTGTTGAATTTTCG |
| *At**Hsp101* | *At1G74310* | F: TGCATTTAGCTGGTGCTTTGAT  R: CCACCGGCACTAGAGATTGC |
| *At**Hsa32* | *At4G21320* | F: GGAAGAGTTTCGAGGAGAACGA  R: GACCTCGCATCTCCGTAACAC |
| *AtHsfA3* | *At5g03720* | F: CCAAGGAATTTCAAACACAACA  R: TCGTTAGCGAATTCCCACTT |
| *At**APX2* | *At3G09640* | F: TCAGGATTCGAGGGTGCATG  R: AAGGCATCCTCATCTGCAGC |
| *At**LEA14* | *At1G01470* | F: ACCGGATTTAATTCATTAAGCGCT  R: TCCCAAGCTGGCAGAGGGAAT |
| *At**RD29A* | *At5G52310* | F: TGGATCTGAAGAACGAATCTGATATC  R: GGTCTTCCCTTCGCCAGAA |
| *AtRD29B* | *At5G52300* | F: AAACGGCTACCACATCCAAG  R: CCTCCAATGGATCCTCGTTA |
| *At**ABI5* | *At2G36270* | F: AAATTGCCGAAATCGAACAG  R: TTTTCCCTCGTTCTGTCTCC |
| *AtActin2* | *At3g18780* | F: TCCCTCAGCACATTCCAGCAGAT  R: AACGATTCCTGGACCTGCCTCATC |
| *GFP* |  | F: CGCACCATCTTCTTCAAGGAC  R: ACGTTGTGGCTGTTGTAGTTGT |
| *NtUbiquitin* |  | F: TCCAGGACAAGGAGGGTAT  R: CATCAACAACAGGCAACCTAG |

Supplemental Information Table S3. Primers used for plasmid reconstruction.

| *pCAMBIA1300* | *p1300-LlDREB2B* | F: GCTCTAGAATGGTGAAGCAAGAGGACTCGGAGGT  R: GGGGTACCCTAGAACAAGCTCCCTCCCTGCAAAGC |
| --- | --- | --- |
| *pCAMBIA1300-C-GFP* | *p1300-LlDREB2B-GFP* | F: GCTCTAGAATGGTGAAGCAAGAGGACTCGGAGGT  R: GGGGTACCCTAGAACAAGCTCCCTCCCTGCAAAGC |
| *pCAMBIA1300-C-GFP* | *p1300-LlDREB2B-△NLS-GFP* | F1: GCTCTAGAATGGTGAAGCAAGAGGACTCGGAGGT  R1: ATGGCAATGGACATTCCGGGTTGCCTTCTCACTTTC  F2: GAGAAGGCAACCCGGAATGTCCATTGCCATTATCGTG  R2: GGGGTACCCTAGAACAAGCTCCCTCCCTGCAAAGC |
| *pCAMBIA1300-C-GFP* | *p1300-LlDREB2B-D-GFP* | F1: GCTCTAGAATGGTGAAGCAAGAGGACTCGGAGGT  R1: TATTCCATCTCTATCTCCACTTGGGATGTTGAGTCGTGC  F2: AACATCCCAAGTGGAGATAGAGATGGAATAAGCAGCTCA  R2: GGGGTACCCTAGAACAAGCTCCCTCCCTGCAAAGC |
| *pCAMBIA1391-GUS* | *p1391-proLlDREB2B-GUS* | F: AACTGCAGTGGAGGGTTTCGATCGATTATGAG  R: CGGGATCCCTCCGAGTCCTCTTGCTTCACCAT |
| *pGBKT7* | *pGBKT7-LlDREB2B* | F: CGGGATCCGATGGTGAAGCAAGAGGACTCGGA  R: CGGGATCCGATGAGGGGAAAAGGAGGCCCTGA |
| *pGBKT7* | *pGBKT7-AtDRIP1*  *125-C end* | F: CGGAATTCAGAACTAAAGCACCTACAAGAAAAG  R: AACTGCAGTTACATGTTGCACTCTGGTAGCTTCC |
| *pGBKT7* | *pGBKT7-AtDRIP2*  *131-C end* | F: CGGGATCCGTAAAAGAACAAAAGCTGCTACGAGAAAAGATG  R: AACTGCAGTTAGTTGTTGCATTCAGGGAGCTTCCGAGA |
| *pGBKT7* | *pGBKT7-AtBPM2* | F: CATGCCATGGAGATGGACACAATTAGGGTTTCCAAGGA  R: CGGGATCCCTAATGTAACCGTTGCTTCACACGTCT |
| *pGBKT7* | *pGBKT7-LlBPM2* | F: CGGAATTCATGGGCGTTG TCCGGGCTTGCAGG  R: GCGTCGACTTATATCTTGGGCTTGACACGCCTTCCA |
| *pGBKT7* | *pGBKT7-AtRCD1* | F: CGGAATTCATGGAAGCCAAGATCGTCAAGGTGT  R: GCGTCGACTTACAATCCACCTGCACCTTCTTCATGGT |
| *pGBKT7* | *pGBKT7-LlRCD1* | F: CGGAATTCATGCGCCTTAGTGATCTTTCAATAAC  R: CGGGATCCCTAGTCTAGCGGTGCATCAGAGT |
| *pGADT7* | *pGADT7-AtDREB2A* | F: CGGAATTCATGGCAGTTTATGATCAGAGTGGAGA  R: CGAGCTCTTAGTTCTCCAGATCCAAGTAACTCAAG |
| *pGADT7* | *pGADT7-LlDREB2B* | F: CGGGATCCATATGGTGAAGCAAGAGGACTCGGAG  R: CCGCTCGAGCTAGAACAAGCTCCCTCCCTGCAAAGC |
| *pGADT7* | *pGADT7-LlDREB2B*  *1-300* | F: CGGGATCCATATGGTGAAGCAAGAGGACTCGGAG  R: CCGCTCGAGCCTACCTCGTCGTCGGATCAAGGAATTCA |
| *pGADT7* | *pGADT7-LlDREB2B*  *1-275* | F: CGGGATCCATATGGTGAAGCAAGAGGACTCGGAG  R: CCGCTCGAGCCTACAGCATCTTCGCATCCAGATTTTGCT |
| *pGADT7* | *pGADT7-LlDREB2B*  *1-245* | F: CGGGATCCATATGGTGAAGCAAGAGGACTCGGAG  R: CCGCTCGAGCCTAAGCTCCATTTTCCTGATCAGTTTC |
| *pGADT7* | *pGADT7-LlDREB2B*  *1-215* | F: CGGGATCCATATGGTGAAGCAAGAGGACTCGGAG  R: CCGCTCGAGCCTATCCGAAAGAGTTTTCGGACTTCCCCA |
| *pGADT7* | *pGADT7-LlDREB2B*  *1-200* | F: CGGGATCCATATGGTGAAGCAAGAGGACTCGGAG  R: CCGCTCGAGCCTAGTTGAAAGGAGCATCTGGTGAGCTGCT |
| *pHis2.1* | *pHis2.1-3DRE* | F: CGGAATTCTATACTACCGACATGAGTTCTATACTACCG  ACATGAGTTC TATACTACCGACATGAGTTC  R: GGACTAGTGAACTCATGTCGGTAGTATAGAACTCATGTCGG  TAGTATAGAACTCATGTCGGTAGTATA |
| *pHis2.1* | *pHis2.1-3mDRE* | F: CGGAATTCTATACTAAAAAAATGAGTTCTATACTAAAA  AAATGAGTTC TATACTAAAAAAATGAGTTC  R: GGACTAGTGAACTCATTTTTTTAGTATAGAACTCATTTT  TTTAGTATAGAACTCATTTTTTTAGTATA |
| *pHis2.1* | *pHis2.1-3B-DRE* | F: CGGAATTCAGCCATCGGTTGATGAGGCCCTTTGGTTATCGG  ATAACTAAAGTCGGTTGTGGTC  R: GGACTAGTTACATACCGAAGAGTTGGCATATTGGGGACCACA  ACCGACTTTAGTTATCCGATAAC |
| *pHis2.1* | *pHis2.1-3B-mDRE* | F: CGGAATTCAGCCAAAAAATGATGAGGCCCTTTGGTTATCGGA  TAACTAAAAAAAAATGTGGTC  R: GGACTAGTTACATTTTTTAGAGTTGGCATATTGGGGACCA  CATTTTTTTTTAGTTATCCGATAAC |

Supplemental Information Table S4. Analysis of putative regulatory *cis*-elements of *LlDREB2B* promoter.

| **Putative *cis*-element** | **Signal sequence** | **Function** | **Location** |
| --- | --- | --- | --- |
| ARR1AT | NGATT | cytokinin-regulated transcription factor，ARR1-binding element | 14(+),43(+),49(-),95(-),111(+),118(+),351(+) |
| CAATBOX1 | CAAT | responsible for the tissue specific promoter activity | 48(+),109(-),49(-),113(-),132(+),144(-),162(+) |
| GTGANTG10 | GTGA | Late pollen gene expression | 51(-),60(+),356(+),368(+),558(-),583(+) |
| CACTFTPPCA1 | YACT | mesophyll-specific，C4 phosphoenolpyruvate carboxylase | 52(+),137(-),154(+),190(-),319(+),1211(-) |
| MYBCORE | CNGTTR | Involved in regulation of genes that are responsive to water stress | 66(+),281(+),1169(-) |
| WBOXNTERF3 | TGACY | May be involved in activation of ERF3 gene by wounding; | 76(-),478(+),592(-),906(+),915(+),973(+) |
| MYCCONSENSUSAT | CANNTG | Dehydration-responsive gene rd22; regulates the transcription of CBF/DREB1genes in the cold | 319(+),372(-),1077(-) |
| MYCATERD1 | CATGTG | MYC recognition sequence necessary for expression oerd1 (early responsive to dehydration) | 1108(+) |
| IBOXCORE | GATAA | Light regulation | 121(-),178(+),693(+) |
| MYB1AT | WAACCA | Dehydration-responsive | 104(-),771(-) |
| GT1GMSCAM4 | GAAAAA | Salt-induced SCaM-4 gene expression | 843(-) |
| DOFCOREZM | AAAG | binding of Dof proteins and PBF | 168(-),188(+),446(+),513(+),683(-),794(-) |
| DPBFCOREDCDC3 | ACACNNG | ABA response | 342(-),363(-),1077(-),1108(-) |
| MYCATRD22 | CACATG | ABA signaling;  dehydration-responsive | 1108(-) |
| INRNTPSADB | YTCANTYY | Light-responsive transcription | 820(+),1241(-) |
| ABREATCONSENSUS | YACGTGGC | ABA-responsive element(ABREs) | 1032(-),1075(-) |
| CAREOSREP1 | CAACTC | GA-responsive element(GAREs) | 412(-) |
| GATABOX | GATA | Required for high level, light regulated,and tissue specific expression | 122(-),178(+),192(-),257(+),259(-)，693(+),701(-),764(+),931(+) |

Supplementary Information Table S5 Analysis of conserved DRE of HsfA3 promoter from different species.

| **Species** | **Gene** | **Gene ID** | **Location and sequence** |
| --- | --- | --- | --- |
| *Oryza sativa* | *OsHsfA3* | Os02g32590 | **-784**-GTCGGT-**-779** |
| *Zea mays* | *ZmHsfA3* | NM_001154496.1 | **-210**-GTCGGC-**-205** |
| *Solanum lycopersicum* | *SlHsfA3* | NM_001247925.2 | **-900**-ACCGAC-**-895** |
| *Elaeis guineensis* | *EgHsfA3* | NC_025993.1 | -**852**-GTCGGT-**-847**  **-99**-GTCGGC-**-94**  **-70**-GTCGGT-**-65**  **-27**-ACCGAC-**-22** |
| *Arabidopsis thaliana* | *AtHsfA3* | At5g03720 | **-305**-GTCGGT-**-300**  **-138**-GTCGGT-**-133** |
| *Lilium longiflorum* | *LlHsfA3B* |  | **-706**-GTCGGT-**-701** |
