# Supplementary material for: A Canonical DREB2-Type Transcription Factor in Lily Is Post-translationally Regulated and Mediates Heat Stress Response
Source: Front Plant Sci. 2018 Mar 8;9:243. doi: 10.3389/fpls.2018.00243 (PMC5852537; doi:10.3389/fpls.2018.00243)
Supplement: Supplementary file 2 [file Image_1.PDF]

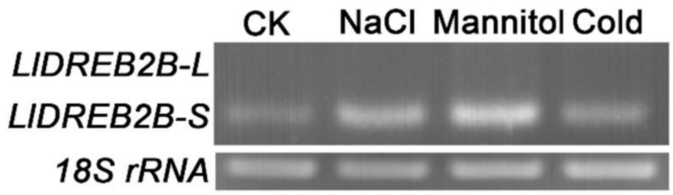

Figure S1 RT-PCR assay of transcript accumulations of *LlDREB2B-L* and *LlDREB2B-S* under cold, salt, and mannitol treatments. For cold treatments, tissue-cultured lily seedlings were treated for 24 h at 4°C. For salt and mannitol treatments, lily seedlings had their roots in water (CK), salt solution (NaCl, 200 mM), or mannitol solution (400 mM) for 24 h. Lily *18S rRNA* was used for data normalization. Each treatment included three plants. Bands were confirmed by sequencing. Three independent experiments were performed, and one representative is shown.

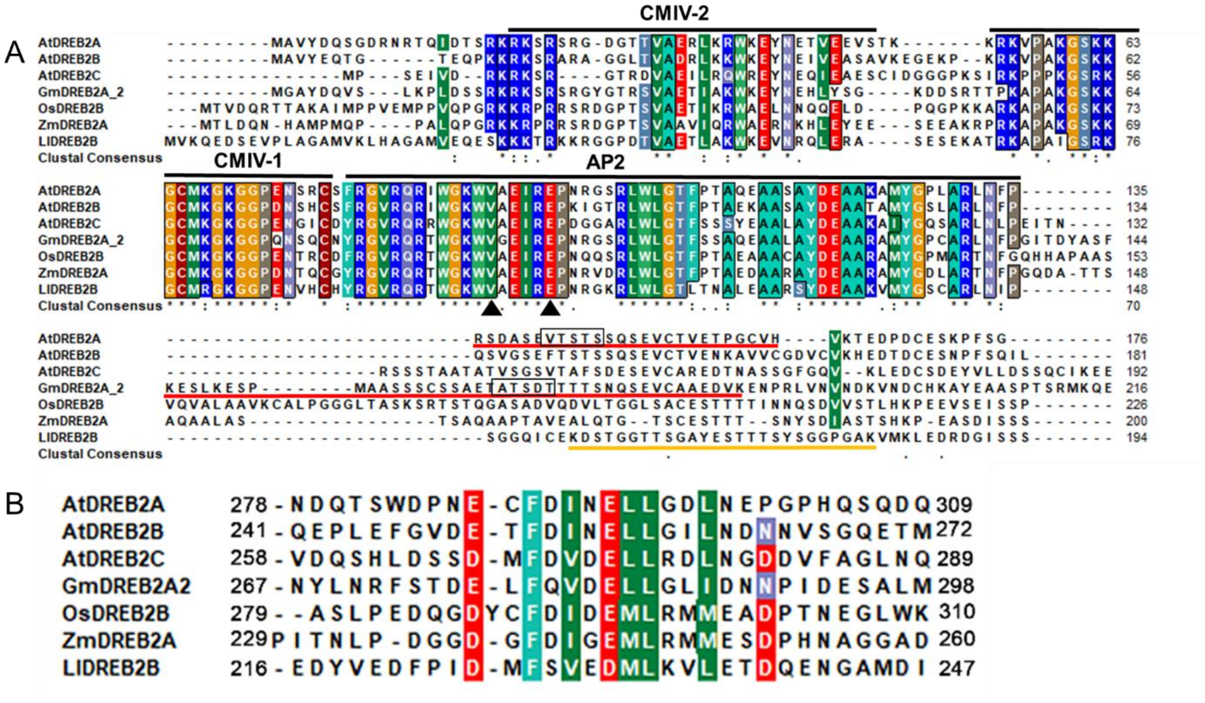

Figure S2 Alignment and schematic diagram of LlDREB2B and homologous proteins. (A) Alignment of the LlDREB2B amino acid sequence with six typical DREB2-type proteins from soybean, Arabidopsis, rice, and maize, performed using BioEdit7.0 software. Color shading indicates identical and conserved amino acid residues. Single black lines represent predicted motifs (CMIV-1, CMIV-2, and AP2). Single red lines represent the NRD of AtDREB2A and GmDREB2A2, respectively. The single orange line represents the predicated NRD of LlDREB2B (http://emboss.bioinformatics.nl/cgi-bin/emboss/epestfind). Black boxes indicate the SBC and SBC-like motifs. Black triangles represent the V14 and E19 conserved amino acids of the AP2 domain. Accession numbers are shown in supplementary Figure S3. (B) The predicated CMIV-3 motifs are referenced to Nakano et al. (2006).

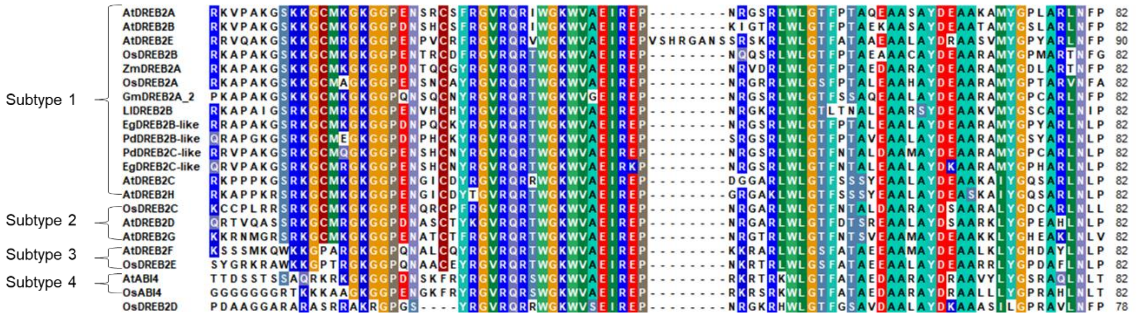

Figure S3 Alignment of the DNA-binding domain and the N-terminal conserved region of DREB2-type transcription factors from different plants. The four subtypes of DREB2-type transcription factors are indicated. Locus identifiers are as follows: AtDREB2A (*Arabidopsis thaliana*, At5g05410), AtDREB2B (*Arabidopsis thaliana*, At3g11020), AtDREB2C (*Arabidopsis thaliana*, At2g40340), AtDREB2D (*Arabidopsis thaliana*, At1g75490), AtDREB2E (*Arabidopsis thaliana*, At2g38340), AtDREB2F (*Arabidopsis thaliana*, At3g57600), AtDREB2G (*Arabidopsis thaliana*, At5g18450), AtDREB2H (*Arabidopsis thaliana*, At2g40350), AtABI4 (*Arabidopsis thaliana*, At2g40220); OsDREB2A (*Oryza sativa*, LOC_Os01g07120), OsDREB2B (*Oryza sativa*, LOC_Os05g27930), OsDREB2C (*Oryza sativa*, LOC_Os08g45110), OsDREB2D (*Oryza sativa*, LOC_Os05g39590), OsDREB2E (*Oryza sativa*, LOC_Os03g07830), OsABI4 (*Oryza sativa*, LOC_Os05g28350); GmDREB2A_2 (*Glycine max*, Glyma14g06080); EgDREB2B-like (*Elaeis guineensis*, LOC105044742), EgDREB2C-like (*Elaeis guineensis*, LOC105052245); PdDREB2B-like (*Phoenix dactylifera*, LOC103696434), PdDREB2C-like (*Phoenix dactylifera*, LOC103714369).

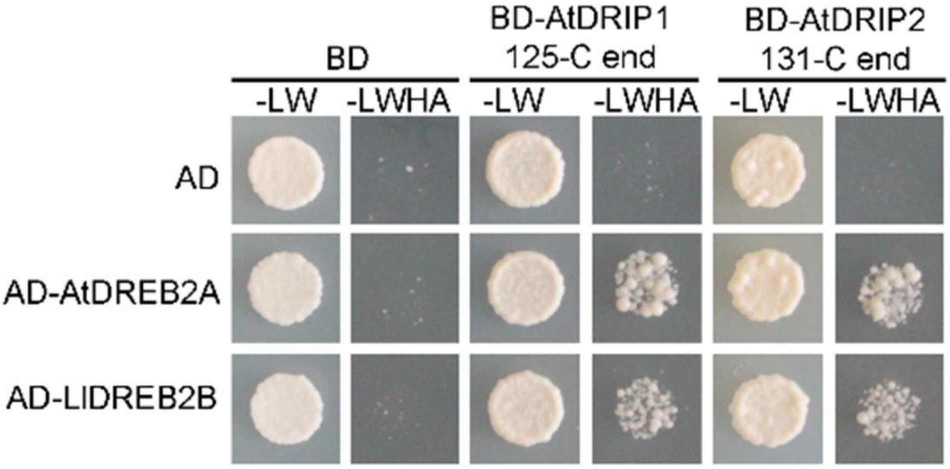

Figure S4 AtDRIP1 and AtDRIP2 interact with LlDREB2B. Interaction of LlDREB2B with AtDRIP1 and AtDRIP2 determined by yeast cell growth on SD medium lacking Leu, Trp, His, and Ade (SD-LWHA).

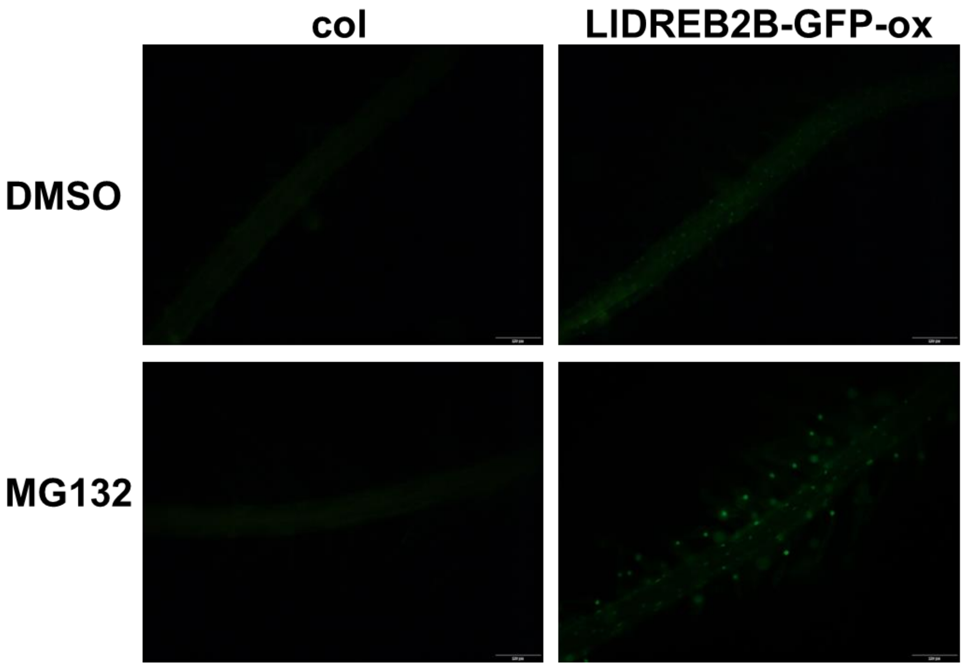

Figure S5 Proteasome inhibitor MG132 treatment of *LlDREB2B-GFP* transgenic plants. The wild-type and *LlDREB2B-GFP* transgenic plants were treated with 50 μM DMSO (mock treated) or 50 μM MG132 for 12 h under dim light conditions and observed under a confocal microscope. Bar = 200 μm.
